# The impact of conscientiousness, mastery, and work circumstances on subsequent absenteeism in employees with and without affective disorders

**DOI:** 10.1186/s40359-017-0179-y

**Published:** 2017-03-29

**Authors:** Almar A. L. Kok, Inger Plaisier, Johannes H. Smit, Brenda W. J. H. Penninx

**Affiliations:** 1 Department of Sociology, VU University Amsterdam, Amsterdam, The Netherlands; 2 Department of Epidemiology & Biostatistics, VU University Medical Center, Amsterdam, The Netherlands; 3 The Netherlands Institute for Social Research, The Hague, The Netherlands; 4 Department of Psychiatry, Amsterdam Public Health research institute, VU University Medical Center, Amsterdam, The Netherlands

**Keywords:** Depression, Anxiety, Absenteeism, Personality, Work

## Abstract

**Background:**

High numbers of employees are coping with affective disorders. At the same time, ambitiousness, achievement striving and a strong sense of personal control and responsibility are personality characteristics that are nowadays regarded as key to good work functioning, whereas social work circumstances tend to be neglected. However, it is largely unkown how personality characteristics and work circumstances affect work functioning when facing an affective disorder. Given the high burden of affective disorders on occupational health, we investigate these issues in the context of affective disorders and absenteeism from work. The principal aim of this paper is to examine whether particular personality characteristics that reflect self-governance (conscientiousness and mastery) and work circumstances (demands, control, support) influence the impact of affective disorders on long-term absenteeism (>10 working days).

**Methods:**

Baseline and 1-year follow-up data from 1249 participants in the Netherlands Study of Depression and Anxiety (NESDA) in 2004–2006 was employed. Multivariate logistic regression analyses were performed, including interaction effects between depressive, anxiety, and comorbid disorders and personality and work circumstances.

**Results:**

In general, mastery and conscientiousness increased nor diminished odds of subsequent long-term absenteeism, whereas higher job support significantly decreased these odds. Interaction effects showed that the impact of affective disorders on absenteeism was stronger for highly conscientious employees and for employees who experienced high job demands.

**Conclusions:**

Affective disorders may particularly severely affect work functioning of employees who are highly conscientious or face high psychological job demands. Adjusting working conditions to their individual needs may prevent excessive work absence.

## Background

A substantial proportion of the work force suffers from depression and anxiety disorders (e.g. 6.4% of US workers with a Major Depressive Disorder [1]; in the Netherlands 9 and 7% of workers with an anxiety or depressive disorder respectively [2]). One work outcome that is particularly affected by such disorders and has substantial individual and societal impact is absenteeism. Depression, anxiety, and burn-out are associated with exceptionally long spells (up to 55 days on average) of absenteeism from work [3–5].

At the same time, ambitiousness, achievement striving and a strong sense of personal control and responsibility are highly valued individual characteristics in contemporary Western societies. Governments and employers appear to regard such characteristics as key to good work functioning and successful employability [6–8]. This call for self-governance for instance entails the requirement that employees take individual responsibility for their professional career by seeking new challenges, formulating and striving towards ambitious goals, and constantly ‘work on themselves’ in order to retain their employability and profitability [6, 9]. It is questionable, however, whether employees who embody such characteristics have better work outcomes, and it is largely unknown whether they respond differently to affective disorders from those whose personalities less strongly reflect self-governance. Moreover, an emphasis on self-governance in the workplace may downplay the importance of work circumstances [7, 9], such as psychological demands, social support and control over work, whose effects on work functioning have been shown in numerous studies [10–12].

Given the scarcity and inconclusiveness of prospective research in this area, this paper aims to investigate whether personality characteristics that reflect self-governance, and work circumstances buffer or rather increase the impact of affective disorders on work functioning. Since we are interested in characteristics that reflect ‘self-governance’, this study focuses on two particular personality characteristics of which we will argue that they reflect this concept, i.e., conscientiousness [13] and mastery [14]. Following the widely applied Job Demands-Control-Support model, we include psychological job demands, job control, and social support [15] as work circumstances. Furthermore, we focus on absenteeism as a key indicator of work functioning.

### Previous studies on personality characteristics that reflect self-governance

Aspects of conscientiousness are competence, orderliness, dutifulness, achievement striving, self-discipline and deliberation [13]. Judge et al. ([16], p. 747), describe conscientious persons as “purposeful, strong willed, determined, punctual and reliable”. As such, of the “Big Five” personality characteristics, we argue this aspect of personality most closely resembles one’s disposition towards self-governance. Research on the relationship between conscientiousness and absenteeism has produced mixed results.

A cross-sectional study using data from the Netherlands Study of Depression and Anxiety [17] showed that for employees with depressive or anxiety disorders, higher conscientiousness was associated with lower odds of having had long-term absenteeism (more than two work weeks) in the previous 6 months, and for employees without depressive or anxiety disorders, it was associated with lower odds of short-term absenteeism (1 day up to two work weeks). On the other hand, Johns [18] found in a cross-sectional study that conscientiousness was not significantly associated with absence from work, and Detrick, Chibnall and Luebbert [19] found that orderliness, one dimension of conscientiousness, predicted longer rather than shorter subsequent periods of absenteeism. In one longitudinal study, higher conscientiousness predicted less subsequent absenteeism, but only after adjustment for previous absenteeism [16].

In addition to conscientiousness, *mastery* may constitute a second individual characteristic that clearly reflects a sense individual control over individual (work) outcomes. Mastery is defined as “the extent to which one regards one’s life chances as being under one’s own control in contrast to being fatalistically ruled” ([14]; p. 5). The concept is akin to *locus of control*, coined by Rotter [20]. If a person has internal locus of control, the sense of mastery is high, reflecting the feeling that one is personally responsible for and capable of influencing one’s life outcomes. In contrast, external locus of control reflects the feeling that forces outside oneself, e.g. other people, fate or ‘society’, determine one’s life course [14].

A meta-analysis [11] found that internal locus of control was significantly related to several work outcomes, such as higher job satisfaction, lower turnover intention and lower job stress and burnout. However, about 90% of the included studies were cross-sectional, providing little evidence for a possible causal effect of mastery on such outcomes. Prospective studies found that higher mastery predicted greater ease of reemployment [21] and better job performance [22], but studies on absenteeism are scarce. A cross-sectional study found that for employees with depression and anxiety disorders, higher mastery was associated with lower odds of long-term (but not short-term) absenteeism, while for employees without affective disorders, higher mastery was related to lower odds of short-term (but not long-term) absenteeism [17]. On the basis of available empirical evidence, we expect higher mastery to predict less subsequent absenteeism.

Bono and Judge [23] found that conscientiousness and mastery are moderately correlated (*r* = .31). This finding supports the expectancy that conscientiousness and mastery are partly similar characteristics, but also that they have distinct features that may complement each other. While mastery reflects general feelings of control over life outcomes, conscientiousness reflects a particular way in which individuals strive to accomplish these life outcomes.

### Previous studies on work circumstances

Conscientiousness and mastery are considered as characteristics that are relatively stable over time, and thus strongly bound to the individual. In contrast, work circumstances strongly depend on others. A widely used model for describing the relationships between work circumstances and work functioning is the Job Demands-Control-Support model [15, 24]. Psychological job demands reflect the psychological or mental workload, as well as experienced “organization constraints on task completion and conflicting demands” ([15]; p. 323). Job control – or *decision authority* – is defined as “the worker’s control over the performance of his or her own job” (ibid., p.323). Job control includes not only the level of skill and creativity needed to perform the job, but also the extent to which employees experience freedom in choosing the way in which they execute their work. Job support reflects the amount of social support that is experienced from coworkers and supervisors, and also identifies the presence of conflicts at work.

Plaisier et al. [25] found that particularly high job support, high job control and reduced working hours were cross-sectionally associated with better work functioning and less absenteeism. This equally applied to employees with and without a depression or anxiety disorder. However, no impact of job demands on absenteeism was found. A meta-analysis by Michie and Williams [26] covered a large variety of work factors and work outcomes. The review includes ten studies on absenteeism. These studies showed that higher job support (two studies) and higher control (seven studies) tend to decrease absenteeism. Perhaps surprisingly, higher demands (two studies) also decreased absenteeism. Results were roughly the same for cross-sectional and longitudinal or experimental studies, although some cross-sectional studies had null findings.

In summary, the evidence on the relationships between conscientiousness and mastery and absenteeism is still ambiguous. To the contrary, most studies on work circumstances indicate that higher support and control associated with less absenteeism. For job demands no clear pattern was found. Moreover, few studies investigated whether the impact of affective disorders on absenteeism might be different for those with different personality or work circumstances. We aim to reveal to what extent personality characteristics that reflect achievement striving and control, and work circumstances affect the impact that developing an affective disorder has on subsequent absenteeism. Specifically, we address the following research question: to what extent do conscientiousness, mastery, and job demands, control, and support affect the relationship between depressive and anxiety disorders and absenteeism?

By addressing this question, this study strengthens empirical evidence on how emphasizing individual self-governance and personal responsibility in the workplace may affect work functioning, particularly of psychologically vulnerable employees. Results may also inform mental health practitioners and specialists in occupational rehabilitation about which individual and work-related factors are most fruitful to intervene on, given the psychological and psychopathological profile of employees.

## Methods

### Data and sample

Data was gathered from the Netherlands Study of Depression and Anxiety. NESDA aims to investigate the long-term course of depression and anxiety disorders, in order to extend scientific knowledge and improve prevention and treatment programmes. NESDA includes Major Depressive Disorder (MDD), Minor Depression, Dysthymia, Generalized Anxiety Disorder (GAD), Social Phobia, Agoraphobia, and Panic Disorders. In 2004, 2981 respondents aged 18–65 years old were recruited via primary care practices (*n* = 1610), earlier studies in the Netherlands (NEMESIS and ARIADNE; *n* = 564), and mental practices and hospitals (*n* = 807), making the sample representative for people within different health care settings and developmental stages of psychological problems. A total of 1701 respondents had a current (6-month recency) depressive and/or anxiety disorder, 2329 respondents (additionally) had a lifetime diagnosis, and 652 respondents had no current or lifetime diagnosis [27].

Information on demographics, personality characteristics, work circumstances, psychological wellbeing, physical health as well as genetical and neurological information was obtained through face-to-face interviews, telephone interviews and medical examinations. Through this multidisciplinary approach, insights from psychosocial and biological research paradigms can be integrated. The study protocol has been approved by the Medical Ethical Review Board of the VU University Medical Centre, and all participants provided written informed consent. More detailed information on NESDA can be found in [27].

In the current study, all independent variables were assessed in the baseline interview. For the dependent variable, data from a 1-year follow-up self-report questionnaire was used. The sample selection procedure for the present study was as follows. From the 2981 baseline participants, respondents who were employed for at least 12 h per week at baseline were selected as the initial study sample (*n* = 2003). This included respondents with partial sickness benefit or partial occupational disability who still worked more than 12 h a week. Freelancers and respondents on pregnancy leave were excluded. Subsequently, respondents who did not participate in the follow-up measurement (*n* = 352), did not (completely) answer questions on work circumstances and personality characteristics, became unemployed or worked less than 12 hours a week, or did not report the amount of absenteeism at 1-year follow-up, were excluded from the initial study sample (*n* = 754 in total). The statistical analyses are therefore based on 1249 respondents, which represents 62% of the initial study sample.

### Operationalization

#### Long-term absenteeism

The amount of absenteeism in the year after baseline was assessed by the question “Have you been absent from work in the previous year due to health problems, and if so, for how many working days?”. Eleven respondents mentioned extremely long periods of absenteeism (over 260 days). These values were limited to 260 working days (52 weeks * 5 working days a week). Respondents were not asked to distinguish between partial and full day sickness absence.

Because the sample distribution of absenteeism was skewed, absenteeism was dichotomized. Following Plaisier et al. [25], a cut-off point of 11 or more working days of absenteeism was used for indicating long-term absenteeism. Two hundred thirty-two respondents met this criterion. It was expected that this categorization would rule out absenteeism caused by common complaints such as the flu or a cold, for which a spell causes 3 days of absence from work on average [28]. Since the focus of this study is on predictors of substantial, long-term absenteeism, it was decided not to include short-term absenteeism as a separate outcome variable in the analyses. Sensitivity analyses using different cut-off points for long-term absenteeism (8 and 15 working days respectively) showed similar results. When using lower or higher cut-off points the impact of the predictors tended to deviate from the impact within the 8–15 working days range.

#### Affective disorders

For descriptive statistics, continuous scales indicating the severity of depression and anxiety symptoms were used. These measures were based on the Inventory of Depressive Symptoms (IDS) for depression severity and the Beck Anxiety Index (BAI) for anxiety severity [27]. For the regression analyses, we used variables expressing the presence of a depression and/or anxiety disorders within the previous 6 months. These diagnoses were assessed by the CIDI interview (Composite International Diagnostic Interview; [29]). Extending the Vlasveld et al. study [17], in the regression models we distinguished three groups: those with a depressive disorder only, those with an anxiety disorder only, and those with comorbidity of depressive and anxiety disorders. Comorbidity in the final sample was as follows: of those with a depressive disorder, 57.5% also had an anxiety disorder, and of those with an anxiety disorder, 52.5% also had a depressive disorder.

The control group consisted of respondents without any affective disorder (*n* = 326), and those without a current, but with a lifetime diagnosis (*n* = 294). We therefore refer to the control group as ‘healthy or lifetime diagnosis’. Although within the control group, those with a lifetime diagnosis scored less favorably on most study variables than those without any diagnosis, these differences were small in comparison with employees with a current disorder.

#### Personality characteristics

From the NESDA-dataset, scores on an abbreviated, 5-item version [30] of the original 7-item Pearlin and Schooler’s Mastery Scale (1978) were used to assess respondents’ level of mastery. Items were answered on a likert-scale ranging from 1 (strongly disagree) to 5 (strongly agree), and included statements such as “I have little control over the things that happen to me”, and “I often feel helpless in dealing with the problems of life”. This scale had high reliability (Cronbach’s α = .88).

The level of conscientiousness was assessed by the NEO-Five Factor Inventory (NEO-FFI) questionnaire, an abbreviated form of the NEO-Personality Inventory (NEO-PI; [13]). Conscientiousness was measured by 12 items answered on a likert-scale ranging from 1 (strongly disagree) to 5 (strongly agree). The total scale score ranged from 12 to 60 points. Example items are “I have a clear set of goals and work toward them in an orderly fashion”, and “I am a productive person who always gets the job done”. Scale reliability in the current sample was high (Cronbach’s α = .80).

#### Work circumstances

For assessing work circumstances, the Job Content Questionnaire (JCQ) [15] was used. We used a Dutch version of the JCQ in which dichotomous items were used (see [31] for details). Three dimensions from this questionnaire were included: psychological job demands (5 items, Cronbach’s α = .76), job support (8 items, Cronbach’s α = .82), and job control (or decision authority, six items, Cronbach’s α = .78). Answer categories to the statements were ‘yes’ (1) or ‘no’ (0). The scores on these items were averaged, resulting in a scale range of 0 to 1. Examples of questions for job demands were “Is it hectic at your work?” and “Do you have to work very fast?”. Examples of job support were “Can you appeal to your colleagues when you need to?” and “Are you being sufficiently supported at work by your direct supervisor(s)?”. Examples of job control were “Can you decide for yourself how to execute your work?” and “Can you decide to interrupt your work any time you wish to?”.

#### Covariates

The analyses were controlled for a number of demographic variables, for chronic diseases, and for previous absenteeism. Since an extensive literature exists that shows structural differences in psychopathology between men and women (e.g. [32]), gender of the respondent was added as a control variable. Research in the Netherlands also shows that younger and higher educated persons structurally exhibit less absenteeism than older and lower educated persons [5]. Therefore, the analysis was controlled for age and years of education. Since it is likely that the presence of chronic diseases may explain a share of absenteeism [33], the number of chronic diseases was added as a covariate. In NESDA, this was assessed by a count of the number of self-reported somatic conditions consisting, including heart diseases, diabetes, stroke, arthritis, cancer, hypertension, intestinal problems, liver disease, epilepsy, chronic lung problems, allergy and injuries. This variable ranged from 0 to 8.

We adjusted the analyses for previous absenteeism. This was self-reported as the number of absence days in the 6 months preceding the baseline interview. Values exceeding 130 working days (26 weeks * 5 working days), were limited to 130 days.

### Statistical analyses

Independent sample t-tests and chi-square tests were performed to explore differences between respondents who were included versus excluded from the initial study sample (*n* = 2003). Furthermore, differences between respondents with and without current depressive, anxiety and comorbid disorders within the final sample were estimated (*n* = 1249).

Logistic regression models were employed to estimate odds of long-term absenteeism during 1-year of follow-up, as predicted by the independent variables. All independent variables except dichotomous ones were standardized. First, the separate impact of the predictors was investigated in two models that adjusted for different sets of control variables. Second, a multivariate analysis was performed in which all variables were simultaneously added. Third, we tested in total eighteen interaction effects within eight different models (two personality characteristics and three work circumstances * three dummies for affective disorders in five separate models, and three interactions among the work circumstances in three separate models). Interaction effects were considered statistically significant at the *p* < .05-level.

## Results

### Descriptive statistics

The 1249 included respondents were older and higher educated than the excluded respondents (Table 1). Additionally, the included had significantly better physical and mental health at baseline, as indicated by having fewer chronic diseases, less severe depressive symptoms, and less severe anxiety symptoms. There were also statistically significant differences in mastery and conscientiousness between the included and excluded group, although absolute differences were small. Differences in work circumstances at baseline were small or non-existent, but previous absenteeism was much lower in the included than in the excluded sample.

**Table 1:** Descriptive statistics of the initial (*N* = 2003) and final study sample (*N* = 1249) ^a^

	Initial sample ^b^				Final sample		
	Total (working at t0)	Excluded	Included	*p*-value	Depr. and/or anx.	Healthy or lifetime	*p*-value
	*N* = 2003	*N* = 754	*N* = 1249	excl-incl	*N* = 629	*N* = 620	
Socio-demographics							
% female	65.3	63.8	66.2	.27	66.9	65.5	.59
Age [18–65]	41.0 (11.9)	39.9 (12.6)	41.6 (11.3)	.002	41.0 (10.7)	42.2 (11.9)	.06
Education in years [5–18]	12.5 (3.2)	12.0 (3.2)	12.9 (3.2)	<.001	12.6 (3.3)	13.2 (3.1)	<.001
No.of chronic diseases [0–7]	0.8 (1.0)	0.9 (1.1)	0.7 (0.9)	.002	0.8 (0.9)	0.7 (0.9)	.02
Number of working hours	29.7 (12.1)	28.2 (13.9)	30.5 (10.8)	<.001	30.4 (10.5)	30.6 (11.0)	.76
Depression and anxiety							
Severity of depression [0–85]	20.2 (13.7)	23.0 (14.2)	18.5 (13.1)	<.001	26.8 (11.8)	10.2 (8.1)	<.001
Severity of anxiety [0–63]	11.3 (10.2)	13.2 (11.0)	10.1 (9.5)	<.001	15.3 (9.8)	4.9 (5.4)	<.001
% Depressive disorder only	14.7	15.7	14.1	.34	28.0	n/a	
% Anxiety disorder only	17.6	18.3	17.2	.54	34.2	n/a	
% Comorbid disorder	23.7	31.4	19.1	<.001	37.8	n/a	
Personality characteristics							
Mastery [5–25]	17.7 (4.4)	17.1 (4.5)	18.0 (4.4)	<.001	15.9 (4.1)	20.2 (3.5)	<.001
Conscientiousness [12–60]	42.2 (6.4)	40.9 (6.6)	43.0 (6.2)	<.001	41.3 (6.3)	44.6 (5.6)	<.001
Work circumstances							
Job demands [0–1]	0.47 (0.34)	0.44 (0.35)	0.49 (0.34)	.03	0.50 (0.35)	0.47 (0.33)	.21
Job control [0–1]	0.75 (0.30)	0.72 (0.32)	0.76 (0.30)	.07	0.71 (0.31)	0.79 (0.28)	<.001
Job support [0–1]	0.70 (0.30)	0.70 (0.30)	0.70 (0.30)	.71	0.64 (0.31)	0.76 (0.28)	<.001
Absenteeism							
% long-term absenteeism [>10 days subsequent year]	18.4	20.0	18.2	.55	23.9	12.4	<.001
Absence previous 6 months [0–130 working days]	15.2 (31.3)	19.5 (36.4)	12.5 (27.5)	<.001	20.4 (34.5)	4.6 (13.8)	<.001
Absence during 1-year follow-up [0–260 working days]	10.5 (29.5)	10.5 (23.7)	10.5 (30.2)	.99	15.1 (38.1)	5.9 (18.3)	<.001

^a^ Numbers within [] are ranges, numbers within () are standard deviations

^b^ Excluded are employees who were not employed anymore at t1 and/or had missing data on absenteeism, personality characteristics and/or work circumstances

Within the final sample, 28% of respondents with affective disorders had a depression only, 34.2% had an anxiety disorder only, and 37.8% had a comorbid disorder. Respondents with affective disorders reported lower mastery and conscientiousness than respondents without current affective disorders (*n* = 620; *t* = −19.92, *p* < .001 and *t* = −9.65, *p* < .001 respectively). Furthermore, they experienced less job control and less job support (*t* = −4.67, *p* < .001 and *t* = −7.09, *p* < .001 respectively), but did not differ in reported psychological job demands (*t* = −1.26, *p* = .21).

The percentage of respondents reporting long-term absenteeism during follow-up was much higher in the group with a current depressive and/or anxiety disorder than in the group without a current disorder (23.9% versus 12.4%; $\chi$ ^2^ = 27.6, *p* < .001). Expressed in working days, those with a current disorder reported two-and-a-half to four times longer absenteeism during the 6 months before baseline (*t* = 15.83, *p* < .001), and in the year after baseline (*t* = 9.25, *p* < .001) than those without a current disorder. In general, absenteeism during follow-up was much shorter than before baseline, which might be explained by the fact that the respondents at baseline had recently suffered from an affective disorder or were still suffering, and the symptoms will probably have diminished during follow-up, generally resulting in less absenteeism.

### Bivariate analyses

Separate effects of the independent variables, adjusted for different sets of control variables, are presented in Table 2. Model 1 shows that, adjusted for demographics and chronic diseases, having a current depressive and comorbid disorder significantly increased odds of subsequent long-term absenteeism (*Odds Ratio* (*OR*) = 3.19, *p* < .001 and *OR* = 2.35, *p* < .001 respectively). Employees with only an anxiety disorder had no significantly higher odds of long-term absenteeism than those without any disorder. Higher mastery predicted lower odds of long-term absenteeism (*OR* = 0.79, *p* = .002), and the impact of conscientiousness in this model was non-significant. Higher job demands predicted higher odds of long-term absenteeism (*OR* = 1.16, *p* < .05), while higher job control (*OR* = 0.81, *p* < .05) job support (*OR* = 0.74, *p* < .001) decreased odds of long-term absenteeism.

**Table 2:** Logistic regression of long-term absenteeism during 1-year follow-up on separate predictors (*n* = 1249) ^a^

	Model 1 *adjusted for gender, education, age, chronic diseases*		Model 2 *additionally adjusted for previous absenteeism*	
	Odds Ratio	*p*-value	Odds Ratio	*p*-value
Affective disorders (ref = no)				
Current depressive disorder	3.19	<.001	2.62	<.001
Current anxiety disorder	1.37	.16	1.24	.35
Current comorbid disorder	2.35	<.001	1.73	.01
Personality characteristics				
Mastery	0.79	.002	0.88	.11
Conscientiousness	0.91	.22	0.98	.75
Work circumstances				
Job demands	1.16	.045	1.14	.08
Job control	0.81	.01	0.84	.02
Job support	0.74	<.001	0.79	.002

^a^ All independent variables except dichotomous ones are standardized

Model 2 additionally adjusted for previous absenteeism, which explained a substantial part of the relationships of the other variables with absenteeism. The effects of mastery and job demands became non-significant, while the effects of depressive and comorbid disorders, job control, and job support weakened but remained statistically significant.

### Multivariate analysis

In model 3 (Table 3) all predictors were simultaneously added. *N* decreased to 1222 due to complete case analysis. The variables jointly accounted for 12% of the variance in subsequent long-term absenteeism. Employees suffering from a depressive or comorbid disorder had higher odds of long-term absenteeism than those without a disorder (*OR* = 2.55, *p* < .001 and *OR* = 1.74, *p* < .05 respectively), and there was no effect of having only an anxiety disorder. Neither mastery nor conscientiousness had a statistically significant effect on the risk of long-term absenteeism. The level of job demands and job control were unrelated to long-term absenteeism, while higher job support significantly decreased odds of long-term absenteeism (*OR* = 0.83, *p* < .05).

**Table 3:** Logistic regression of long-term absenteeism during 1-year follow-up on all predictors (*n* = 1222)^a^

Model 3	Odds Ratio	*p*-value	95% C.I.
Covariates			
Gender	0.93	.64	0.67–1.28
Education	0.91	.25	0.78–1.07
Age	1.23	.02	1.04–1.45
Chronic diseases	1.05	.53	0.90–1.23
Previous absenteeism	1.40	<.001	1.23–1.61
Affective disorders (ref = no)			
Current depressive disorder	2.55	<.001	1.62–4.02
Current anxiety disorder	1.31	.26	0.82–2.09
Current comorbid disorder	1.74	.02	1.08–2.81
Personality characteristics			
Mastery	1.08	.44	0.89–1.31
Conscientiousness	1.07	.46	0.90–1.26
Work circumstances			
Job demands	1.03	.70	0.88–1.21
Job control	0.88	.11	0.75–1.03
Job support	0.83	.02	0.70–0.97
*Nagelkerke R Square*	.12		

^a^ All independent variables except dichotomous ones are standardized

### Interaction effects

In five models, interaction effects between the dummies for affective disorders and each of the two personality characteristics and three work circumstances were estimated. We found that the effect of an anxiety or comorbid disorder on absenteeism was stronger for highly versus less conscientious employees (*OR* = 2.05, *p* < .01 and *OR* = 1.61, *p* < .05 respectively; Table 4). Specifically, we calculated that highly conscientious (+1 SD) employees with an anxiety or comorbid disorder had respectively 2.31 and 2.65 times higher odds of long-term absenteeism compared to highly conscientious employees without a current affective disorder. In contrast, employees with average conscientiousness suffering from an anxiety or comorbid disorder had only 1.13 and 1.65 times higher odds of long-term absenteeism than those without a disorder with the same level of conscientiousness. Thus, highly conscientious employees appear to be more vulnerable to anxiety and comorbid disorders than their less conscientious counterparts.

**Table 4:** Results from models with significant interaction effects between affective disorders and personality or work circumstances ^a^

	Odds Ratio	*p*-value	95% C.I.
Interaction model 1. Affective disorders x conscientiousness			
Main effect depressive disorder	2.35	<.001	1.50–3.69
Main effect anxiety disorder	1.13	.63	.70–1.82
Main effect comorbid disorder	1.65	.04	1.02–2.65
Main effect conscientiousness	0.75	.045	.56–.99
Depressive disorder x conscientiousness	1.58	.06	.99–2.51
Anxiety disorder x conscientiousness	2.05	.003	1.27–3.31
Comorbid disorder x conscientiousness	1.61	.03	1.06–2.47
Interaction model 2. Affective disorders x job demands			
Main effect depressive disorder	2.47	<.001	1.55–3.93
Main effect anxiety disorder	1.32	.24	.83–2.11
Main effect comorbid disorder	1.74	.03	1.07–2.84
Main effect job demands	0.82	.13	.63–1.06
Depressive disorder x job demands	1.67	.03	1.07–2.62
Anxiety disorder x job demands	1.42	.13	.91–2.21
Comorbid disorder x job demands	1.33	.15	.90–1.96

^a^ Variables not shown in the table are: gender, education, age, chronic diseases, previous absenteeism, conscientiousness (only model 2), job demands (only model 1), mastery, job support, job control. All independent variables except dichotomous ones are standardized

We found a similar pattern for job demands and depressive disorders. The impact of a depressive disorder (but not an anxiety or comorbid disorder) on long-term absenteeism was stronger for employees with higher job demands than for employees with lower job demands (*OR* = 1.67, *p* < .05). Specifically, employees with high job demands (+1 SD) who faced a depressive disorder had 4.12 times higher odds of long-term absenteeism compared to those with high job demands but no current disorder, while the Odds Ratio was 2.47 in employees with average job demands. Employees with high job demands are thus more vulnerable to depressive disorders than their counterparts with lower job demands.

Finally, we also tested interaction effects between the three work circumstances, but none of them reached statistical significance.

## Discussion

We have empirically addressed the question whether characteristics that reflect individual achievement striving and control prospectively predict work absence up and above the effects of work circumstances that greatly depend on cooperation with others. Furthermore, we have assessed to what extent mastery, conscientiousness, and work circumstances buffer or rather increase the effects of anxiety, depressive, and comorbid disorders on subsequent long-term absenteeism. By controlling for previous absence, our analysis captures the ‘long arm’ of affective disorders, regardless of earlier absenteeism that may have been related to these disorders.

Largely contradicting the thesis that individual achievement striving and control are key to good work functioning, we found that mastery and conscientiousness were in general not associated with (lower) risks of subsequent long-term absenteeism. For work circumstances, we found that higher job support significantly decreased risks of long-term absenteeism, regardless of affective disorders. Moreover, analyses of interaction effects provided the key findings of this paper. The impact of affective disorders on absenteeism differed between employees with different personality and work circumstances. Anxiety and comorbid disorders had more severe effects on absenteeism in employees with higher conscientiousness, and depressive disorders had more severe effects in employees with higher job demands. In terms of absenteeism, these findings thus identified employees who are highly conscientious and who experience high psychological job demands as particularly vulnerable to affective disorders.

Our findings on conscientiousness seem to contradict previous cross-sectional research by Vlasveld et al. [17], who showed that higher conscientiousness might be protective for absenteeism both in employees with and without depressive or anxiety disorders. This discrepancy might be explained by the fact that in the earlier study the diagnosis of the mental disorder took place at an unspecified moment during the preceding 6 months, while absenteeism was based on the entire previous 6 months. Therefore, the detrimental effects of the combination of high conscientiousness and an affective disorder may not yet have been observed for those employees in which the disorder manifested only shortly before the interview. By controlling for previous absenteeism, the current study rules out this possibility. Moreover, we distinguished three forms of affective disorders (depression only, anxiety only, and comorbidity), specifying in more detail how personality and work factors may influence the impact of particular psychological conditions on absenteeism from work.

Furthermore, it has been demonstrated that highly conscientious employees may experience greater decreases in well-being after becoming unemployed than those who are less conscientious. This is possibly because ‘failure’ is experienced more negatively in those who strongly feel that they should be reliable and personally responsible for successful functioning at work [34]. Since feelings of failure also often accompany affective disorders, this may explain the extreme negative impact of anxiety and comorbid disorders in those who are highly conscientious. It has also been found that persons high in self-control tend to be relied on more often and more heavily by co-workers, which makes them experience a greater “burden of responsibility” on the job [35]. This suggests that severe mental problems may impede highly conscientious workers’ capability to bear this responsibility, possibly leading to more absenteeism from work. Such interactive mechanisms might explain the contradictory findings from previous research on the relationships between conscientiousness and work functioning.

### Limitations

A strong feature of the present study is the longitudinal data, allowing assessment of the impact of three patterns of affective disorders, personality characteristics, and work circumstances on future absenteeism, while controlling for previous absenteeism. Nevertheless, some limitations should be discussed to properly qualify the findings.

First, it may be argued that low mastery and conscientiousness are symptoms of affective disorders, rather than independent of them. The correlations between affective disorders and mastery and conscientiousness were moderately strong, but no problems with multicollinearity were found. Therefore, the regression models accurately take the overlap into account. The impact of mastery and conscientiousness on long-term absenteeism may therefore be regarded as being independent of affective disorders. To the extent that conscientiousness and affective disorders were mutually interdependent, this was demonstrated through their interaction effects.

Second, the interpretation of ‘personality’ is widely debated. Costa & McCrae [13] prefer the interpretation that personality characteristics reflect “the view the individual has of him- or herself” (ibid., p.8). It may therefore be argued that such questionnaires do not measure objective personality. However, such measures of personality are in practice unavailable, or an objective personality may not exist. Moreover, it is shown that personality characteristics, as measured by the NEO-FFI questionnaire, are stable over time, and as such they seem reliable predictors of various outcomes [13, 36]. Similar to the NEO-FFI, the Job Content Questionnaire [15] is based on self-reports, and therefore contains a certain amount of subjectivity. However, this does not disqualify the predictive value of these widely validated measures for work-related outcomes such as absenteeism.

Third, ‘work functioning’ is a broad concept, and we have only partly captured this by focusing on absenteeism as an outcome. Although some cross-sectional studies have been conducted (e.g., [25]) future studies could focus on presenteeism and the associated productivity loss while working with an affective disorder [37].

A final issue is the relatively healthy condition of the respondents included in the study sample compared to the respondents who were excluded on the basis of various criteria. Almost half of the excluded group consisted of respondents who did not participate in the follow-up measurement, which may be explained by the tendency of people with impaired (mental) health to drop out of longitudinal research. This might also partly explain the relatively low number of absence days, even in the group with affective disorders. Additionally, part of the excluded sample reduced working hours from more to less than twelve hours a week between waves, which may have been due to deteriorating health. Therefore, the strength of the effects found in this study may have been underestimated in comparison to a wider population of employees.

## Conclusion

The present study showed that in general, one’s personal disposition towards achievement striving and personal responsibility and control had few effects on long-term absenteeism, while high social support reduced absenteeism in our overall sample. Moreover, highly conscientious employees and employees who experience high psychological job demands appeared to be particularly at risk for long-term absenteeism when developing an affective disorders. This suggests that particularly those employees who highly value individual achievement, endorse strong norms of personal responsibility, or have psychologically demanding work might get caught in a counterproductive circle of increasing work absence when faced with psychological problems. Our study may inform employers, occupational rehabilitation specialists, and mental health practitioners that although anxiety and depressive disorders are generally detrimental for work functioning, these employees may be particularly vulnerable. Perhaps counterintuitive to some, an appeal to their conscientious character, or sense of personal responsibility for successful employability may be counterproductive. Lowering demands and increasing social support might be better strategies.
